# A Unique Phenotype of Maturity-Onset Diabetes of the Young With a Novel Disease-Causing Insulin Gene Variant

**DOI:** 10.1210/jcemcr/luae230

**Published:** 2024-12-23

**Authors:** Cherie Chua, Clara Si Hua Tan, Su Chi Lim, Rashida Farhad Vasanwala

**Affiliations:** Department of Paediatric Endocrinology, Kandang Kerbau Women's and Children's Hospital, 229899 Singapore; Clinical Research Unit, Khoo Teck Puat Hospital, 768828 Singapore; Diabetes Centre, Khoo Teck Puat Hospital, 768828 Singapore; Department of Medicine, Khoo Teck Puat Hospital, 768828 Singapore; Department of Paediatric Endocrinology, Kandang Kerbau Women's and Children's Hospital, 229899 Singapore

**Keywords:** maturity-onset diabetes of the young, pediatric diabetes mellitus, insulin gene, insulin resistance

## Abstract

Maturity-onset diabetes of the young (MODY) represents 1% to 5% of patients with diabetes mellitus (DM), and numerous genes associated with MODY have been identified. While mutations of the insulin gene (*INS*) are known to cause permanent neonatal DM, rare disease-causing variants have also been found in MODY. These patients demonstrate variable clinical phenotypes—from milder forms requiring lifestyle or oral agent interventions to severe forms requiring lifelong insulin. We present a case of MODY arising from a novel disease-causing *INS* variant, in an adolescent with atypical features. He was obese with clinical evidence of insulin resistance, diagnosed with DM through opportunistic oral glucose tolerance testing. He developed symptomatic hyperglycemia with worsening glycemic trend, requiring treatment with high-dose insulin and metformin. After 2.5 years, his glycemic profile normalized following weight loss, and pharmacotherapy was discontinued. Targeted gene testing revealed a de novo novel missense variant in exon 2 of the *INS* gene (p.His29Tyr), confirmed using bidirectional Sanger sequencing. Insulin resistance in patients with MODY can worsen their clinical course and increase risks of long-term complications. Management of these patients should be individualized. This case highlights the utility of genetic testing in diagnosing uncommon and variable forms of MODY, particularly those with atypical features.

## Introduction

The changing face of childhood diabetes mellitus (DM) over the past decade has made classification of a newly diagnosed patient more challenging [1]. Factors resulting in the overlapping clinical features of different subtypes of DM (type 1 [T1DM], type 2 [T2DM] and monogenic diabetes) include the global rise in prevalence of obesity and increasing disease awareness, hence earlier presentation and greater beta cell reserves at diagnosis [2, 3]. Advancements in diagnostics and treatment are paving the way for precision medicine and personalized management of patients with DM.

MODY is the most common type of monogenic diabetes, representing 1% to 5% of all patients with DM [4]. Patients with MODY are a genetically and clinically heterogenous group, and establishing the diagnosis warrants an index of suspicion. Typical features include young onset of hyperglycemia (before 25 to 30 years of age), absence of diabetes autoantibodies, absence of insulin resistance, residual beta cell function that persists during follow-up, and family history of early-onset diabetes in a first-degree relative [5]. The actual prevalence of MODY is likely to be underestimated, as patients may be misclassified as T2DM due to insulin independence, or as T1DM due to young age and normal body mass index (BMI). Treatment of MODY varies from lifestyle modification alone, to the use of oral hypoglycemic agents such as sulfonylureas, while some patients require lifelong insulin [6-11].

Numerous genes have been found to be associated with MODY—including *HNF1A*, *HNF1B*, *HNF4A*, *GCK*, *NEUROD1*, *KLF11*, *PDX1*, *PAX4*, *BLK*, *CEL*, *INS*, *ABCC8*, *KCNJ11*, and *APPL1* [12]. Availability of comprehensive genetic testing has better equipped us to characterize the different types of MODY and guide treatment [13].

*INS*-MODY arises from rare disease-causing variants in the insulin gene (*INS*). While *INS* variants are better known to cause permanent neonatal DM (PNDM), several pathogenic variants have been identified in patients with MODY [14].

In this paper, we describe and discuss the molecular pathophysiology, clinical course, and management of a patient with *INS-*MODY due to a novel de novo variant.

## Case Presentation

**Figure 1.**
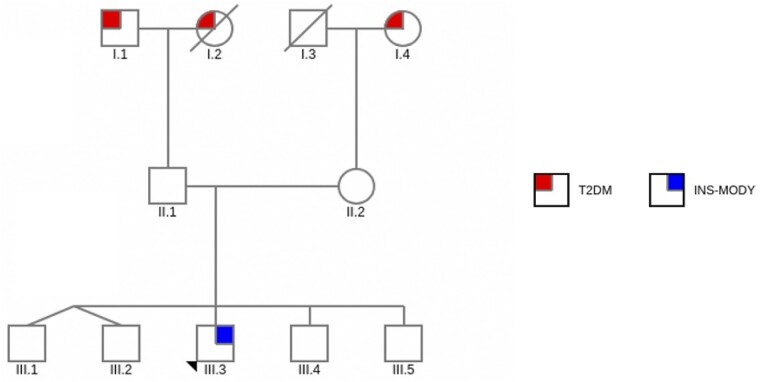
Family pedigree showing history of type 2 diabetes mellitus (T2DM) in affected family members of proband, III.3 (denoted by arrow). Neither of the parents (II.1 or II.2) has a history of DM. The proband's siblings were also unaffected.

Patient M was born full-term with birth weight of 2700 g; he had no maternal gestational diabetes but had family history of T2DM in both paternal and maternal grandparents (Fig. 1). At 10 years 6 months of age, he was admitted to the hospital for gastroenteritis and was noted to be obese. Significant examination findings included generalized obesity (BMI 30.2 kg/m^2^, > 97th centile), mild acanthosis nigricans, and Tanner 2 stage of puberty.

A follow-up screening oral glucose tolerance test (OGTT) was performed the following month: his blood glucose levels were 6.4 mmol/L (at 0 minutes) (normal 3.9-6.0 mmol/L) and 14.7 mmol/L (120 minutes) (normal 3.9-7.7 mmol/L) with glycated hemoglobin (HbA1c) of 6.9% (normal < 6.5%). Lifestyle and dietary modifications were advised. At 3 months after diagnosis, his repeat HbA1c improved to 6.6%, pharmacotherapy was withheld.

## Diagnostic Assessment

He developed symptomatic hyperglycemia 8 months after diagnosis and a repeat HbA1c level rose to 12.9%. Insulin and C-peptide levels were 29.3 mIU/L (203 pmol/L) (normal reference range, 2.40-24.9 mIU/L; 16.7-172.9 pmol/L) and 2.39 µg/L (791 pmol/L) (normal reference range, 1.10-5.00 µg/L; 364-1655 pmol/L), respectively. Autoantibodies for anti-islet cell, glutamic acid decarboxylase, tyrosine phosphatase-related islet antigen 2, and zinc transporter 8 were negative. He was diagnosed as having T2DM.

## Treatment

**Figure 2.**
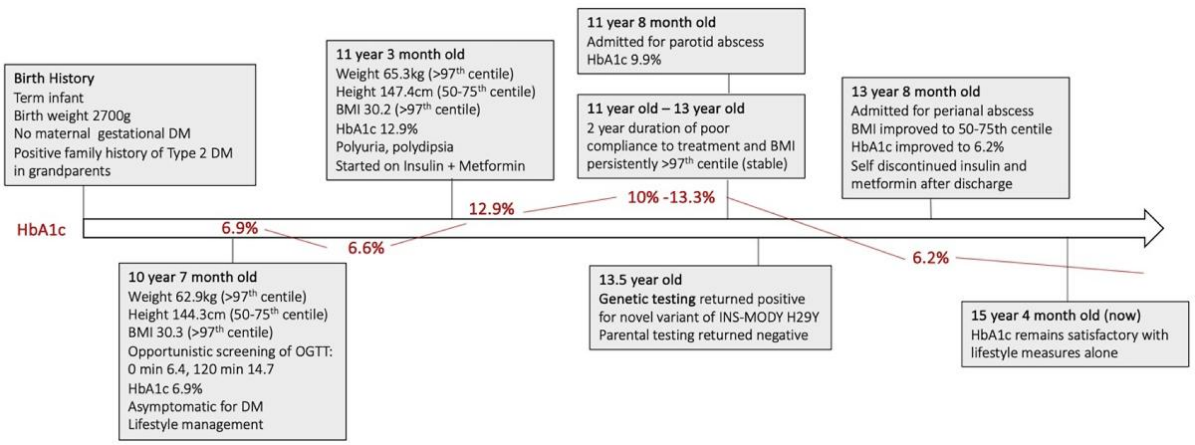
Timeline of patient's clinical course.

The patient was started on metformin (500 mg daily) and insulin (basal-bolus regime, 1 unit/kg/day). Over the subsequent 2 years, HbA1c trended between 10.0% and 13.3%, with poor adherence to insulin and metformin therapy. BMI reduced from > 97th centile to between the 85th and 90th centile. His clinical course was complicated by 2 hospital admissions for parotid abscess and perianal abscess requiring surgical interventions, but he had no hyperglycemic crisis. Insulin and metformin therapies were optimized for better glycemic control (maximum insulin 1.1 units/kg/day, metformin 1 g per day), while the patient continued to work on his lifestyle. His clinical course is demonstrated in Fig. 2.

## Outcome and Follow-Up

With adherence to pharmacotherapy and lifestyle changes, after 2.5 years from diagnosis, his BMI reduced to between the 50th and 75th centile, and his HbA1c improved to 6.2%. The patient subsequently self-discontinued insulin and metformin while continuing with healthy lifestyle measures and was able to maintain satisfactory HbA1c (5.3%-5.8%).

**Figure 3.**
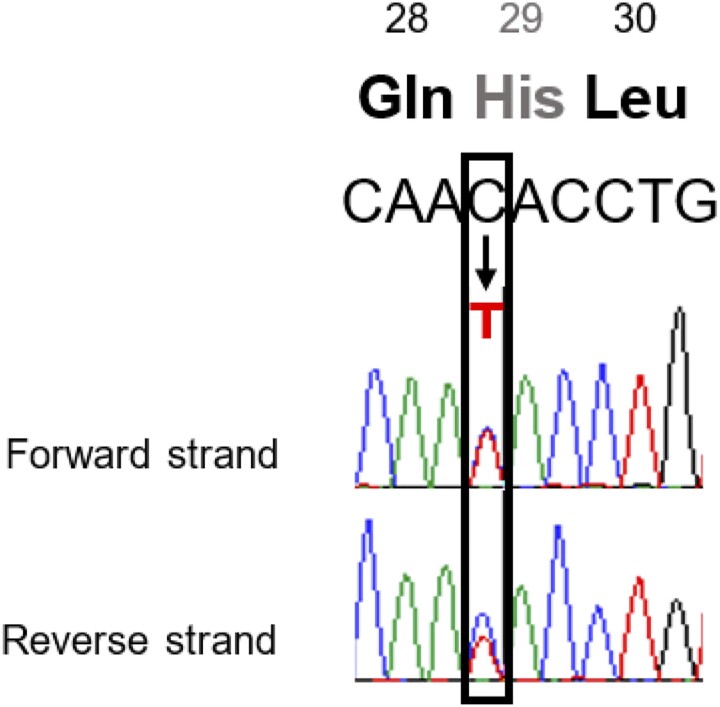
Bidirectional Sanger sequencing chromatogram of a missense variant (p.His29Tyr) in exon 2 of the *INS* gene identified in the proband.

In view of his young age at onset, absence of antibodies, preservation of C-peptide, and having weaned off insulin, he was recruited for genetic testing for MODY using massive parallel sequencing method described [15]. Targeted gene panel sequencing for 16 genes, including major forms of MODY (*HNF1A-, HNF4A-, GCK-* and *HNF1B*-MODY), was performed. Screening for copy number variants in the 4 major MODY-associated genes was also carried out using multiplex ligation probe amplification (MLPA) method. Variants identified were annotated and interpreted using the Alamut Visual Plus software (SOPHiA GENETICS) following American College of Clinical Genetics and Genomics (ACMG) guidelines [16]. The patient was found to carry a novel missense variant [NC_000011.10(NM_000207.3) (*INS*):c.85C>T p.(His29Tyr)] which was absent in population databases (Genome Aggregation Database [gnomAD] and Exome Aggregation Consortium [ExAC]) and had not been reported in the literature before. The presence of the variant was confirmed using bidirectional Sanger sequencing (Fig. 3). The variant is found in exon 2 of the insulin (*INS*) gene (RefSeqGene NG_007114.1) and resides in the B-chain domain, which may affect biosynthesis and processing of insulin [17]. Computational tools (SIFT, PolyPhen-2 Align GVGD, and MutationTaster) predicted the variant to be functionally deleterious. Taken together, the variant is interpreted to be likely pathogenic. Genetic testing carried out in the proband's parents suggested that the variant is de novo.

Patient remains off pharmacotherapy at time of writing, with HbA1c < 6.5%—an unusual progress when compared with other reported *INS*-MODY cases who required insulin.

## Discussion

We present a patient with adolescence-onset diabetes with moderate obesity, negative for multiple autoantibodies and preserved C-peptide levels, who was initially diagnosed T2DM, but subsequent molecular gene testing found *INS*-MODY due to a novel missense variant (p.His29Tyr). Most patients with *INS*-MODY reported in literature are insulin dependent, although with varying degree of insulin requirement. Interestingly, our patient required a relatively high dose of insulin for 2 years, but eventually thrived without insulin, maintaining optimal HbA1c after weight loss.

Variants in the *INS* gene have been reported to cause beta cell dysfunction and were initially found in insulin-dependent patients with PNDM [18, 19]. Variants in *INS* occur rarely in patients with MODY. Recent evidence suggests that the clinical features of *INS*-MODY vary widely, depending on the affected gene locus and resulting disruption [20].

A European study by Edghill et al screened for *INS* variants in 1044 patients with DM diagnosed in infancy, childhood, or adulthood to determine the prevalence and clinical phenotype of *INS*-associated DM [14]. The majority of patients with *INS* variants are diagnosed with diabetes younger than 6 months of age. A novel *INS* variant (NC_000011.10(NM_000207.3) (*INS*):c.16C>T, p.Arg6Cys) was identified as a probable pathogenic variant within a MODY family with 3 affected individuals who were diagnosed at 15, 15, and 65 years old, respectively. All of them had features consistent with a diagnosis of MODY—nonobese and non-insulin-dependent. They were well managed on diet alone for many years before requiring low-dose insulin or oral agents. Another study in 2010 sequenced the *INS* gene in 116 MODY patients of European ancestry and similarly found a novel pathogenic variant (NC_000011.10(NM_000207.3) (*INS*):c.17G>A p.Arg6His)—in the same codon, within a Danish family [12]. While 3 family members (including the proband) were diagnosed with diabetes at 20, 26, and 51 years old, the son of the proband had normal glucose tolerance but reduced beta cell function over 5 years. Affected family members were treated with diet, with or without oral agents. It was postulated that p.Arg6Cys or p.Arg6His substitution, which resides in the signal peptide, results in disruption of the crucial disulfide bond, resulting in misfolded proinsulin molecules accumulating in the endoplasmic reticulum, causing cellular stress and beta cell apoptosis [14, 21].

Another case of *INS*-MODY was described in an Australian family [22]. A heterozygous variant (NC_000011.10(NM_000207.3) (*INS*):c.277G>A p.Glu93Lys) was identified in an 11-year-old patient who presented with mild diabetic ketoacidosis, strong family history of diabetes and low C-peptide, suggesting insulin deficiency. She was treated with increasing insulin doses (0.6 to 0.8 units/kg/day) to maintain HbA1c. Two of her siblings were also diagnosed with diabetes at the age of 8 and were insulin dependent. Her 43-year-old mother was diagnosed at 11 year old with autoantibody-negative T1DM and was insulin dependent, while her 66-year-old grandfather was diagnosed in his thirties with T2DM and remained non-insulin-dependent for 2 decades before transitioning to insulin. The p.Glu93Lys variant was found to segregate in the family in individuals with diabetes. In silico analysis suggested that this variant may result in impaired binding of insulin to its receptor, affecting insulin clearance, increasing half-life and circulating insulin levels [23].

Our patient was diagnosed with diabetes as a preadolescent and presented with insulin resistance due to obesity, adding complexity to his disease etiology. He was treated with metformin and insulin for 2.5 years, which were eventually stopped. His requirement for insulin was likely a result of the compounded effects of MODY and insulin resistance (attributable to presence of background polygenic risk factors) [24, 25]. With subsequent weight loss, his insulin sensitivity improved and insulin dependence reversed. He maintained satisfactory HbA1c levels solely by lifestyle measures, signifying that endogenous insulin production was sufficient. Given that *INS* variants are associated with different mechanisms in the beta cell, causing impaired insulin processing and beta cell failure, it is not unusual that the clinical presentation of *INS*-MODY varies. A more relevant comparison is a proband who presented with insulin-dependent DM diagnosed before 1 year of age, who was found to carry an *INS* variant within the same codon—p.His29Asp, which was shown to disrupt folding and conformation of proinsulin in vitro, affecting stability of insulin [8]. We postulate that nonsynonymous variants resulting in alterations of the amino acid at position 29 of the *INS* gene can reduce the protein's thermodynamic stability and result in varying degree of endoplasmic reticulum stress. This is also supported by the molecular mechanism of rapid-acting insulin analogue Lispro, developed by inversion of proline and lysine at positions 28 and 29 of the insulin polypeptide chain B respectively, resulting in a change in pharmacokinetics [26]. Along similar principles, endogenous alterations of the amino acid at position 29 of the *INS* gene could affect molecular dynamics.

*INS* variants remain a rare cause of MODY and affected individuals present with heterogenous clinical phenotypes, making an accurate diagnosis challenging [27]. We recommend genetic testing for patients suspected to have MODY or when etiology of DM is unclear; suggestive features include young onset, absence of beta cell autoantibodies, low insulin requirement, persistent measurable C-peptide levels, isolated renal glycosuria with or without renal abnormalities, and persistent hyperglycemia without ketoacidosis in patients with T1DM; or hyperglycemia without obesity, acanthosis nigricans, insulin resistance, and normal triglyceride and low-density lipoprotein in patients with T2DM [5].

Genetic testing may be performed sequentially or comprehensively at once depending on the costs and available resources. As *GCK*, *HNF1A*, and *HNF4A* variants make up almost 80% of all MODY cases, it may be prudent to test for these first [11]. Testing for the less common genes can be carried out if the initial test is unyielding. Establishing a genetic diagnosis will help physicians better characterize each type of MODY, guide therapeutic decisions, and avoid unnecessary insulin. The variable expressivity of the disease-causing variants means there is no one-size-fits-all approach and that management still requires individualization. However, definitive molecular diagnosis followed by in vitro functional assays to elucidate the underlying molecular mechanisms may aid development of new drugs or treatment strategies for this unique group of patients. This knowledge may help in prognostication of the disease course and influence screening for vascular complications [28].

## Learning Points

- We describe an atypical case of *INS*-MODY due to a novel de novo variant, c.85C>T p.(His29Tyr), confounded by presence of obesity-driven insulin resistance.
- Patients with *INS*-MODY are clinically heterogenous and personalized treatment should be recommended based on individual's disease etiology, with consideration of both monogenic and polygenic risk factors.
- A patient with MODY and obesity-driven insulin resistance, who is insulin dependent, can potentially be weaned off insulin with weight loss and reversal of the insulin resistance. Lifestyle interventions should be emphasized in this group of patients to avoid unnecessary insulin treatment and reduce risk of long-term vascular complications.
- Genetic testing for MODY should be considered in patients with atypical features and unclear etiology of DM. Characterization of patients with MODY in relation to their genotype may help us develop better treatment strategies and prognostication.

## Contributors

All authors made contribution to authorship. C.S.H.T., S.C.L., and R.F.V. contributed to the diagnostic process; C.C. and R.F.V. were involved in patient management and manuscript submission. All authors reviewed and approved the final draft.

## Data Availability

Original data generated and analyzed during this study are included in this published article.

## Abbreviations

BMI: body mass index
DM: diabetes mellitus
HbA1c: glycated hemoglobin
MODY: maturity-onset diabetes of the young
PNDM: permanent neonatal diabetes mellitus
T1DM: type 1 diabetes mellitus
T2DM: type 2 diabetes mellitus
